# Exploiting Generative Design for 3D Printing of Bacterial Biofilm Resistant Composite Devices

**DOI:** 10.1002/advs.202100249

**Published:** 2021-05-29

**Authors:** Yinfeng He, Meisam Abdi, Gustavo F. Trindade, Belén Begines, Jean‐Frédéric Dubern, Elisabetta Prina, Andrew L. Hook, Gabriel Y. H. Choong, Javier Ledesma, Christopher J. Tuck, Felicity R. A. J. Rose, Richard J. M. Hague, Clive J. Roberts, Davide S. A. De Focatiis, Ian A. Ashcroft, Paul Williams, Derek J. Irvine, Morgan R. Alexander, Ricky D. Wildman

**Affiliations:** ^1^ Faculty of Engineering University of Nottingham University Park Nottingham NG7 2RD UK; ^2^ School of Engineering and Sustainable Development De Montfort University Leicester LE1 9BH UK; ^3^ Advanced Materials and Healthcare Technologies School of Pharmacy University of Nottingham University Park Nottingham NG7 2RD UK; ^4^ Department of Organic and Medicinal Chemistry School of Pharmacy University of Seville Seville 41012 Spain; ^5^ National Biofilms Innovation Centre University of Nottingham Biodiscovery Institute School of Life Sciences University of Nottingham University Park Nottingham NG7 2RD UK; ^6^ University of Nottingham Biodiscovery Institute School of Pharmacy University of Nottingham University Park Nottingham NG7 2RD UK

**Keywords:** 3D printing, bacterial biofilm resistant, cell instructive, generative design, multi‐material

## Abstract

As the understanding of disease grows, so does the opportunity for personalization of therapies targeted to the needs of the individual. To bring about a step change in the personalization of medical devices it is shown that multi‐material inkjet‐based 3D printing can meet this demand by combining functional materials, voxelated manufacturing, and algorithmic design. In this paper composite structures designed with both controlled deformation and reduced biofilm formation are manufactured using two formulations that are deposited selectively and separately. The bacterial biofilm coverage of the resulting composites is reduced by up to 75% compared to commonly used silicone rubbers, without the need for incorporating bioactives. Meanwhile, the composites can be tuned to meet user defined mechanical performance with ±10% deviation. Device manufacture is coupled to finite element modelling and a genetic algorithm that takes the user‐specified mechanical deformation and computes the distribution of materials needed to meet this under given load constraints through a generative design process. Manufactured products are assessed against the mechanical and bacterial cell‐instructive specifications and illustrate how multifunctional personalization can be achieved using generative design driven multi‐material inkjet based 3D printing.

## Graphical abstract

Multi‐material inkjet‐based 3D printing is used for the design and manufacture of personalized medical devices. A design optimization approach is used to specify the location of two different materials, each with a different moduli, but both with the ability to resist bacterial colonization (up to 75% better than medical silicones), such that they meet user‐defined, complex design constraints.

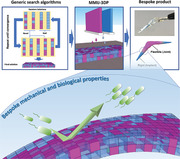

## 1 Introduction

Modern healthcare relies on medical devices, yet a large proportion of patients who receive one can suffer from infection or chronic inflammation that can require antibiotics and corrective surgery. It is becoming increasingly apparent that by selecting appropriate materials, the behavior of attached cells can be controlled, thereby providing a means to designed medical devices with reduced failure rates. Screening libraries of materials has been used to identify polymers with cell‐instructive properties including controlling immune responses, resisting bacterial biofilm formation, promoting stem cell attachment, and the prevention of fungal colonization.^[^ ^1^, ^2^, ^3^, ^4^, ^5^ ^]^ We aim to exploit the materials identified using this approach to achieve multi‐functional medical device production, meeting both cell response requirements and mechanical performance criteria. Here we explored whether this can be achieved using a combination of multi‐material inkjet 3D printing (MM‐IJ3DP)^[^ ^6^, ^7^ ^]^ and genetic algorithms (GA).^[^ ^8^, ^9^ ^]^ The key to this advance is that MM‐IJ3DP allows us to spatially vary the material composition and thus include differentiated functions,^[^ ^10^, ^11^, ^12^ ^]^ while also providing the important scale up capabilities of high resolution and production speeds. This opens the possibility of a new manufacturing concept that allows the user to produce devices with spatially distributed, customizable material functionalities in a cost‐effective manner.^[^ ^13^, ^14^, ^15^, ^16^, ^17^ ^]^

This paper sets out to develop a platform by which MM‐IJ3DP can be used to create bespoke devices with tunable, spatially varying mechanical performance, while incorporating and retaining resistance to bacterial biofilm formation. It has been demonstrated that acrylate based biofilm resistant polymers^[^ ^2^ ^]^ can be formulated for inkjet 3D printing (IJ3DP) with retention of their biofilm resistance, allowing the geometrical freedoms of 3D printing to be employed to create medically‐relevant bespoke products.^[^ ^18^ ^]^ Here we go significantly beyond these two studies by introducing MM‐IJ3DP. In principle, a target geometry can be understood as a cluster of voxels at a user defined size. Through MM‐IJ3DP, the ratio of different inks in each voxel can be engineered by the user and therefore exhibits required performance, for example, modulus, transparency, etc. We developed two inkjet printable biofilm resistant formulations and used a computational design approach to direct the manufacture of multi‐material devices, specifying the deposition location of voxels with different moduli in order to achieve a customized mechanical deformation for a given load. Chemical interrogation of the interface/interphase regions at the junction between different inks demonstrates intimate contact and intermixing between two adjacent materials, resulting in a mechanically robust structure.

This work has been inspired by the need to address device associated bacterial infections and antimicrobial resistance (AMR). Consequently, it avoids the use of antibiotics and other agents which drive the development of antimicrobial resistance and instead, employs bacterial biofilm‐resistant materials that do not kill the bacteria on contact or through leaching. Prevention of bacterial biofilm formation at the surfaces of medical devices is critical. Biofilms are communities of bacteria sequestered within a self‐produced extracellular matrix that achieve up to 1000 times greater tolerance to antibiotics and host immune system defences^[^ ^19^ ^]^ and are a major unsolved global biomedical problem that accounts for 25.6% of all healthcare‐associated infections within the USA alone.^[^ ^20^ ^]^ The incorporation of antibiotics is widely used to reduce device infections, but is often accompanied by localized toxicity,^[^ ^21^ ^]^ active component depletion,^[^ ^22^, ^23^ ^]^ and selection for resistance imposed by the bactericidal nature of most antimicrobial agents^[^ ^24^ ^]^—the materials approach used herein avoids these difficulties. Our advances allow the design and manufacture of highly personalized devices that avoid biofilm formation and, importantly, do not contribute to AMR.

**Figure 1:**
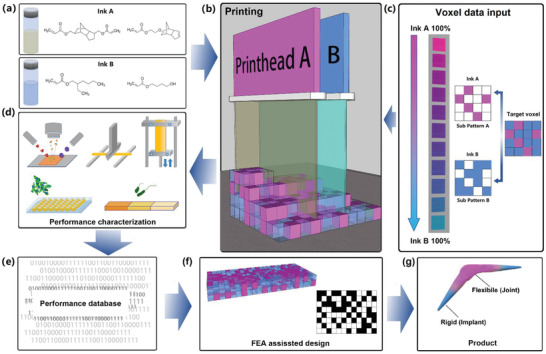
Schematic approach of the methodology followed to develop, 3D print, and characterize bespoke biofilm inhibiting devices. a) Four monomers were chosen from an existing database to obtain two biofilm resistant materials with highly differentiated moduli. b) MM‐IJ3DP was achieved with a dual inkjet printing unit and a UV lamp to trigger the polymerization after ink deposition. c) A pseudo‐randomized printing strategy was used to produce composite voxel with choice of modulus, where complementary sub patterns were generated for ink A and ink B components, where each was the inverse of the other. d) Mechanical test to determine physical properties, ToF‐SIMS to determine chemical composition, and bacterial biofilm inhibition and cell viability assays to assess the physical and biological performances of the MM‐IJ3DP printed devices with proposed ink formulations. e) The performances of specimens with different compositions were collected together to form a database. f) A finite element analysis coupled with a GA was performed to design specimens with the required performance on the basis of the composite properties from the database; g) a device exemplar was manufactured.

We developed two functional ink formulations that inhibit biofilm formation after polymerization while having very different elastic moduli (**Figure** **1**a). Using MM‐IJ3DP we were able to co‐print these two inks to construct composite structures where the volume ratio of each ink was varied to engineer the mechanical performance. Backed by a pseudo‐random co‐deposition polymerization printing strategy, we manufactured complex 3D composite structures with spatially dependent compliance (Figure 1b). The target device was constructed using a user defined “voxel”: each was filled up with a specified volume ratio of ink A and B. Each voxel consisted of 560 pixels (4 pixels in *X*, 4 pixels in *Y* and 35 pixels in *Z*)—sufficient to allow variety in voxel composition and to create voxels of equal length in all three directions. These voxel sizes, however, could be varied to suit the application or resolution requirements as needed. Both inks are randomly distributed within the voxel (Figure 1c) and different volume ratios of the two inks will lead to a variety of voxel properties. We then applied generative design as a tool to decide the location of the different voxels, which led to the manufacture (with MM‐IJ3DP) of structures that met our design requirements (Figure 1f). To confirm if our approach is effective, we carried out in vitro mammalian cell compatibility and bacterial biofilm inhibition tests to learn if the co‐printed composite was still safe and effective; examined the interaction of the sequentially printed materials by high definition time‐of‐flight secondary ion mass spectrometry (ToF‐SIMS) and compared the mechanical performance of the printed device to the model to assure the design was replicated successfully (Figure 1d).

## 2 Results and Discussion

Novel reactive ink formulations were developed and optimized for the MM‐IJ3DP process to produce structures with distinct mechanical performances (rigid or flexible) while possessing resistance to bacterial biofilm formation on the basis of the previously allocated monomer candidate database^[^ ^2^ ^]^ and consideration of molecule flexibility;^[^ ^25^ ^]^ further detail is given in methodology and Supporting Information. Printing was carried out using a dual head printer (PiXDRO LP50, Meyer Burger) assembly with UV lamp (365 nm, 900 mW cm^−2^). Ink A (rigid) was deposited first and pinned by UV, followed by Ink B (flexible) “filling in” the gaps left. A pseudorandom pixel deposition strategy was introduced to avoid pattern replication and minimized any unintended inhomogeneity of material properties (Figure S1, Supporting Information).

### 2.1 Modulus Range of MM‐IJ3DP Printed Cell‐Instructive Composites

**Figure 2:**
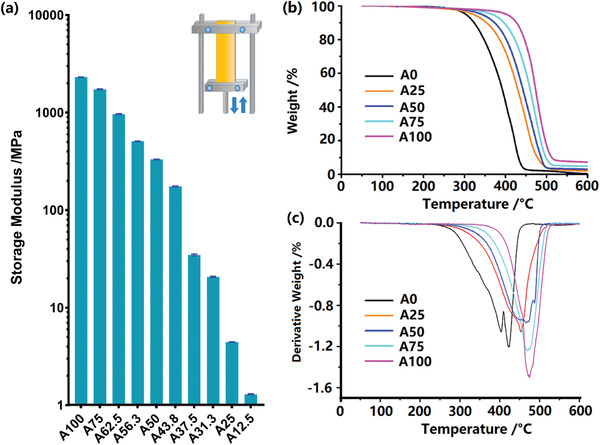
Preliminary assessment of polymer composite specimens with different ratios of ink A and B: a) Dynamic mechanical analysis (DMA) was performed and storage modulus with 10 different compositions were measured (mean ± standard deviation, *n* = 3); b) Thermal gravity analysis (TGA) was carried out for 5 different compositions within a temperature range of 35–600 °C; c) Derivative curve of the TGA to show the decomposition temperature shift for the MM‐IJ3DP printed composites.

It was necessary to determine and codify the relationship between voxel compositions and mechanical properties—this would allow a “dial up” of mechanical composition in the design stage through selection of the balance of ink A and B using our calibration curve. Polymer composite strips (5 × 20 × 1 mm^3^) with 10 different ratios of A to B were produced. The samples were measured using dynamic mechanical analysis (DMA) at 25 °C. **Figure** **2**a shows that the modulus of the polymer composition was engineered by tuning the ratio of ink A and ink B, resulting in an available storage modulus ranging from 1.3 MPa (A12.5) up to 2300 MPa (A100). It was also found that the relationship of storage modulus to the proportion of ink A was nonlinear.

A subsequent study using thermo gravimetric analysis (TGA) was performed to understand further the nature of the printed composite (Figure 2b; Table S1, Supporting Information). Pure Ink A (A100) and Ink B (A0) were tested as control samples, which showed maximum decomposition rate was reached at temperatures of 468 and 414 °C respectively. According to the TGA results from printed composite specimens with 25 v/v% (A25), 50 v/v% (A50), and 75 v/v% (A75) of ink A, the decomposition temperature increased as the proportion of ink A increased. Further derivative analysis of the curve highlighted this trend (Figure 2c). Proportion normalized A100 and A0 curves were subtracted from the correlated composite curves, which revealed a signal hitherto not seen (Figure S2, Supporting Information), which may represent for co‐polymerized molecules of ink A and B formed during the MM‐IJ3DP process.

### 2.2 Bacterial Biofilm Inhibition Assessment of Printed Composites

**Figure 3:**
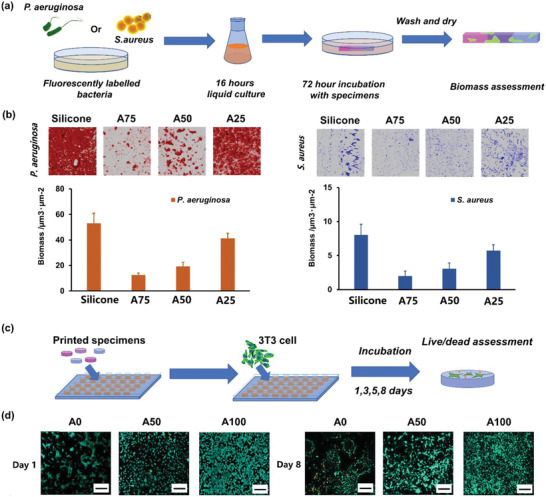
Assessment of bacterial biofilm resistance and mammalian cell biocompatibility of the printed structures: a) Bacterial biofilm formation on the printed sectioned samples containing three compositions(A25, A50, A75) were tested with silicone rubber as a control; b) The biomasses of *P. aeruginosa* and *S. aureus* biofilms were determined after 72 h incubation. Mean ± standard deviation, *n* = 3; each image covers 512 × 512 µm^2^. Fluorescent micrographs of mCherry‐labelled *P. aeruginosa* (red) and GFP‐labelled *S. aureus* (blue) growing on each surface are shown (bottom). c) Live/Dead cell viability assay where live cells were stained with Calcein‐AM (cyan) and dead cells with EthD‐1 (yellow), d) Cell viability when cultured on the top surface of the sample were assessed using Live/Dead assay at Day 1 and Day 8 on A0, A50, and A100 samples (scale bar 200 µm).

A bar specimen (7 × 2 × 2 mm^3^) containing three sections of different compositions (A25, A50, and A75) were printed and incubated with bacteria to assess their biofilm inhibition performance (**Figure** **3**a). The human opportunistic pathogens *Pseudomonas aeruginosa* (gram‐negative) and *Staphylococcus aureus* (gram‐positive) were selected as they are frequently linked with medical device‐associated infections such as those on implanted prostheses and often result in poor clinical outcomes.^[^ ^26^, ^27^ ^]^ Both bacterial strains, tagged with fluorescent proteins, were incubated with the printed specimens for 72 h to allow biofilms to establish. The coverage and biomass of *P. aeruginosa* and *S. aureus* biofilms were quantitatively assessed by confocal fluorescence microscopy. The results revealed that all the printed compositions inhibited biofilm formation for both pathogens in comparison with silicone rubber (Appleton Woods medical grade tubing), a commonly used polymeric biocompatible material for medical devices. Compared with silicone rubber, *P. aeruginosa* showed 76.4 ± 3.0%, 63.4 ± 6.0%, and 21.9 ± 7.4% biofilm biomass reduction on A75, A50, and A25 sections respectively, while for *S. aureus*, the reductions were 75.2 ± 8.7%, 61.7 ± 10.3%, and 28.5 ± 10.7%. As the proportion of ink A increased, the composited material showed greater resistance to biofilm formation. Previous assessments^[^ ^2^ ^]^ indicated that the components of ink A are more effective at resisting colonization than ink B, and thus these observations align with those findings. Ink B, although not as effective as ink A against biofilm formation, still outperforms the current standard (silicone) and thus when used to engineer the desired modulus and flexibility of the printed composite did not substantially degrade the cell‐instructive function. However, when implementing finite element (FE) assisted generative design as a tool, we attempted to constrain the material composition to include ink A as the major component in order to maximize biofilm inhibition.

### 2.3 Mammalian Cell Response Experiments

The potential mammalian cell cytotoxicity of the printed material is a primary consideration for whether a printed device is biocompatible for clinical applications. The evaluation of the in vitro cytotoxicity and cell attachment test were performed by growing immortalized NIH 3T3 mouse embryonic fibroblast cells with material extract (Figures S3 and S4, Supporting Information) and on the material surface (Figure 3c,d) respectively following ISO standard 10993.^[^ ^28^ ^]^ The chosen cells adhered and proliferated on all the samples. Cells presented the lowest values of proliferation on A0 within the testing period and a less uniform distribution. The Live/Dead results confirmed the majority of the cells were viable, presenting an elongated morphology and covering the entire specimen.

### 2.4 Designing Multi‐Material Response through MM‐IJ3DP

The establishment of the relationship between compositions and moduli, combined with the ability to selectively deposit material spatially, enables the user to control the response of an object in a “non‐trivial” way, that is, by varying the distribution of voxels of different compositions, and therefore the modulus, on demand across the object. We demonstrate this capability in two steps: first by showing that it is possible to adjust the location of a flexural region in order to provide a “hinge” in any desired location;^[^ ^29^, ^30^, ^31^ ^]^ second, by using a material optimization approach to show that it is possible to seek a user‐defined beam deformation profile under a fixed loading condition by spatially varying the distribution of composition of the beam only.

**Figure 4:**
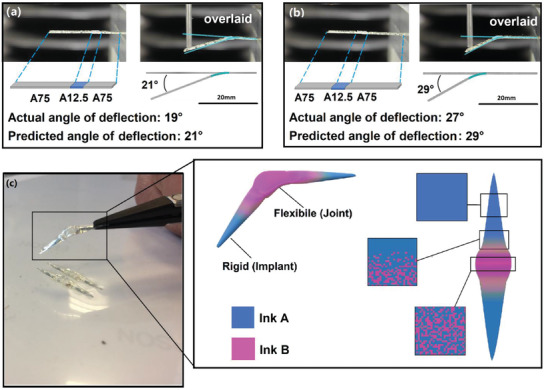
Exemplar of designing the cantilever bending profile by two regions of polymer composites: A75 (grey) and A12.5 (blue) with customized A12.5 (blue) locations, the left edge of the A12.5 region was a) 15 mm and b) 10 mm away from the free end; tests were carried out by applying 5 mm deflection on its free‐end and the predicted deformation obtained from nonlinear FE analysis of the beam is overlaid with experiment data; c) A printed example for a potential application of MM‐IJ3DP printed biofilm resistant medical device: finger joint implant.

In the first instance, we chose a cantilever beam and sought to demonstrate a hinge‐like functionality that could only be achieved when using a homogenous distribution of material by creating a narrow, and consequently weak constriction in the beam. A simple cantilever (25 mm (L) × 5 mm (W) × 0.4 mm (H)) was printed that consisted of 80 v/v% A75 and a short section (20 v/v%) of A12.5 (**Figure** **4**a,b). The “hinge” region (4 mm (L) × 5 mm (W) × 0.4 mm (H)) was shifted by moving the location of the flexible A12.5 segment from 15 mm from the leftmost, that is, free end in Figure 4a to 10 mm from the free end in Figure 4b. The data show that when applying a fixed vertical displacement of 5 mm to the free end, we were able to create a deformation hinged at a specific location by varying the flexible segment location. Such a technique would be applicable to a finger joint prosthetic for example. Current products on the market are “hinged” using a narrow section that allows for a bending motion. However, we also show that this may be achieved through a novel materials composition optimization approach, avoiding the requirement for a thin section and maintaining a bulk section for strength. Further, this device was manufactured using the materials selected for their resistance to bacterial attachment (Figure 4c), an important sought after feature that can reduce bacterial associated infections during and post‐surgery. The use of this material illustrates how other, nonmechanical functionalities can be incorporated into our design and materials selection methodology.

To show the further opportunities for design via material optimization, we developed a computational model of multi‐material structures and assessed them by three‐point bending. This was created in Matlab (R2017a) using an FE model constructed from quadrilateral elements. Each finite element was assumed to be formed from a homogeneous material with a composition varying from A12.5 to A100. Sixteen different possible compositions within this range were chosen and their associated moduli were derived from fitting a curve to the experimental measurement of moduli in Figure 2a. The customized multi‐material sample model used in this study, as a demonstration, was a simply supported beam which was designed to match a pre‐defined deformation profile with a deflection of 0.250 mm at the center when it is subjected to a point load of 2 N. The system was implemented under the constraint of a fixed average material composition (A66), which in this case was inspired by the need to weight the amount of biofilm resistant material in the composite toward ink A (Figure 3a).

**Figure 5:**
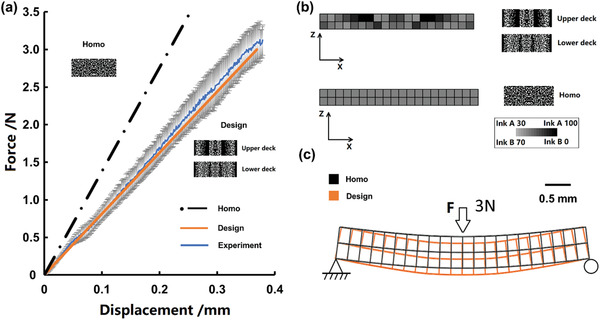
Multi‐material cantilever structure printed with MM‐IJ3DP process: beam specimen printed following the digital design generated from a computational model established in this study: a) Comparison of the mechanical performance between the standard homogeneous cantilever, simulation and experimental (mean ± standard deviation, *n* = 8); b) FEA assisted design of cantilever structure versus homogeneous structure; c) Comparison of deflection between the two cantilevers under 3N loading.

An elastic material model was implemented with an objective function that was composed of the displacement magnitudes, *U*, determined from the Euclidean length of the displacement vector $U\;={\left(u_{x}^{2}+u_{y}^{2}+u_{z}^{2}\right)}^{1/2}$ where *u_x_*, etc. are the individual components of the displacement vector. The objective here was to minimize the root mean square difference between the displacement magnitude of the FE nodes (*U*_FEM_) and the displacement magnitude of corresponding points on an imposed grid on the desired deformation profile (*U*_des_):

(1) $$\mathrm{Minimize}\;\Delta \;=\sqrt{\frac{1}{n}\sum_{i=1}^{n}{\left(U{\left(i\right)}_{\mathrm{FEM}}-U{\left(i\right)}_{\mathrm{des}}\right)}^{2}}$$

where *n* denotes the number of FE element nodes. The objective function defined in the equation above aims to minimize the difference between FE nodal displacements and the displacement of the control points on the desired deformation profile. The displacement magnitudes in this model were calculated from all components of the displacement vector. This was achieved by spatially varying the material composition of finite elements in FE model of the beam iteratively through a GA. A GA was chosen over alternative methods, such as solid isotropic material with penalization,^[^ ^32^, ^33^ ^]^ evolutionary structural optimization,^[^ ^34^ ^]^ and other gradient methods,^[^ ^35^, ^36^ ^]^ as we seek a materials optimization rather than compliance minimization, with the additional benefits that it avoids the need for an objective function that is differentiable, reduces the likelihood of finding local minima and is relatively easy to implement. Convergence of the routine was judged complete when the generation of new designs did not result in behavior different from the previous generation. Upon completion of the iterative steps and convergence of the optimization, our model provided a distribution of material composition corresponding to the elements of the FE model. This predicted distribution of material was tested by replicating this distribution in samples produced using our MM‐IJ3DP approach (5 × 2 × 0.7 mm^3^). These printed samples were tested experimentally using a flexural test fixture^[^ ^37^ ^]^ identical to that used in the model. When the prescribed deflection (0.250 mm) was reached at the center of the bespoke specimen beam, the observed load was 2.12 ± 0.22 N, in agreement with the designed load (2 N). As a comparison, a homogeneous cantilever with the same average composition would deflect by 0.145 mm under the same loading conditions (**Figure** **5**a). The ink A and B distribution in both the homogeneous cantilever and the designed version are presented in Figure 5b, while Figure 5c gives a comparison of their deformation profiles.

### 2.5 Interaction of the Co‐Printed Inks

**Figure 6:**
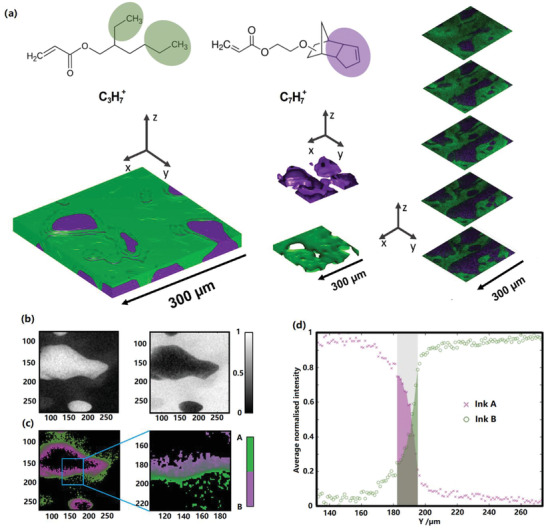
ToF‐SIMS analysis of the interaction between the two printed inks A and B: a) Results showing exclusive characteristic peaks for each formulation and their 3D distribution within an approximate 300 µm × 300 µm × 10 µm volume (ink A in purple and ink B in green, droplet size ≈ 90 µm); b) Intensity distribution of NMF endmembers representing ink A (left) and ink B (right); c) Interface region with intensity between 20% and 80% of the maximum for each ink. Blue rectangle represents area for the *Y*‐axis linescan in d); d) Average intensity distribution inks within the blue rectangle in c). Hashed area represents the interface region.

On the scale of droplets, our fabrication methods do not produce homogenous distributions of ink A mixed with ink B. Instead, they form a composite material made up of distinct and contiguous polymer phases. It is therefore necessary to understand the nature of the interaction at the interface of two dissimilar drops. For this, we exploited ToF‐SIMS 3D analysis to investigate the phases of different inks within the co‐printed composite structure (**Figure** **6**a). Spectra of the individual inks were acquired to identify exclusive signals for each of the formulations via the unsupervised machine learning method “non‐negative matrix factorization” (NMF) of a joint dataset containing spectra of the individual inks as well as spectra of the mixed sample at various depth profiling levels^[^ ^38^ ^]^ (more details in Figure S5, Supporting Information). From NMF endmembers, the secondary ion C_7_H_7_^+^ (91.07 u) was chosen to represent the cyclic structure of monomer EGDPEA of ink A and C_3_H_7_^+^ (43.06 u) to represent propyl end groups of monomer EHA of ink B. Clear separation between ink A (purple) and B (green) was observed in the 3D depth profiling (Figure 6a), with ink B filling up the gaps designed between ink A and covering most of the top surface—a consequence of the sequential printing strategy of printing and pinning drops of ink A, then depositing and curing drops of ink B. Upon further investigation of the spatial intensity distribution of the NMF endmembers representing the two inks, using a 80%/20% definition of the edge spread function^[^ ^39^, ^40^ ^]^ we could determine that the interface (Figure 6b,c) within the observation area has an average width of 16.7 ± 4.3 µm (Figure 6d), which indicates that there was interpenetration during the printing and curing process. The combination of TGA derivative data and ToF‐SIMS analysis of the interface suggests that the interface is composed of both ink A and ink B, predominantly in the form of physical interfacial mixing of molecules of A or B, with the TGA analysis pointing toward the possibility of some chemical copolymerization of ink A and ink B into a new material as a new signal observed (Figure S2, Supporting Information). The emergence of these complex interfacial regions may explain the nonlinear variation of properties such as cytotoxicity (Figure S3, Supporting Information) and modulus (Figure 2) as the proportion of materials A to B is varied.

However, the ToF‐SIMS analysis was not able to resolve the difference between intermixing and co‐polymerization, therefore further investigation is required to conclusively determine the chemical composition of the material at the interface.

## 3 Conclusions

This work has demonstrated the manufacture of MM‐IJ3DP printed devices that are personalisable through generative design guided co‐deposition of inks to create functional composites that are both resistant to bacterial biofilm formation and achieve a specific deformation profile. Our studies showed that it was possible to combine two materials to create a composite that, upon choice of suitable composition ratios, possessed moduli ranging from that of the low modulus material (1.3 MPa) to that of the high modulus material (2300 MPa). We show that using a multi‐material approach guided by a genetic algorithm to decide the precise composition, we can achieve targeted mechanical responses that are not readily accessible when using uniform composite. Inspection of the composite at the droplet level showed that an interphase region formed between the two polymers. In this region we saw chemical evidence of physical intermixing at the molecular level. This creation of a composite of material by design allows the development of sophisticated products where function is distributed throughout the component in a single step of manufacture. Our design tools demonstrated the creation of simple hinges in precise locations as well as deformation profiles that would not be accessible from the homogenous material, under the same loading case. Our combined use of advanced formulations and design tool led MM‐IJ3DP substantially advances our ability to deliver personalized medical devices that have biological and deformation profiles tailorable to individual patient requirements.

## 4 Experimental Section

### Ink Preparation and Assessment

For ink preparation, 20 mL of ink was prepared each time. A corresponding amount of photoinitiator was added in the right combination of monomers and the mixed at room temperature at 800 rpm using PTFE coated magnets (10 mm) till it was fully dissolved. The formulation was then degassed with an N_2_ flow for 15 min.

Monomers and initiator were purchased from Aldrich Chemical Co. and used as received. Viscosities were measured using a Malvern Kinexus Pro Rheometer equipped with a parallel plate at 300 µm separation, under shear rates from 10 s^−1^ and 1000 s^−1^. Each measurement started at 25 °C with 5 °C increments up to 60 °C, the precise increments depending on the sample. A protocol of waiting 300 s after reaching the test temperature was set to ensure the ink was in a steady state condition. At each temperature point and shear rate, the viscosity was recorded at 5 s intervals within a 180 s test time.

### Parameters and Machine Set Up for MM‐IJ3DP

MM‐IJ3DP was performed using a piezoelectric inkjet printer (PiXDRO LP50, Meyer Burger, Figure S8a, Supporting Information) fitted with a dual‐head assembly and two printing heads, each with 128 nozzles (Spectra SE‐128 AA, Fujifilm Dimatix, Santa Clara, USA). The printing temperatures were set at 55 °C for ink A and 25 °C for ink B. The diameter of the nozzles was 35 µm and the nozzle spacing was 508 µm. Heads assembly was equipped with a UV LED radiation source, with a maximum peak at 365 nm. The emission window was 25 mm × 10 mm with an emitted energy of 900 mW cm^−2^. Samples were printed on polyethylene terephthalate (PET) substrate with a set temperature of 25 °C. The whole system was installed inside a glovebox with O_2_ < 300 ppm and a temperature lower than 30 °C. Printing parameters were optimized using Advanced Drop Analysis (ADA) Flexibleware provided by Meyer Burger. Bitmaps were created using Wolfram Mathematica 10.4.

### Material Characterization

To assess the layer thickness for a representative set of formulations, surface profiles were obtained using a Bruker Contour GT‐K Interferometer, equipped with a 5× lens amplified 2×. Vision64TM Flexibleware was used to analyze images, obtaining the values for average height.

Dynamic mechanical analysis (DMA) tests were carried out at room temperature using a PerkinElmer DMA 8000 in tension mode. Specimens were printed following a rectangular pattern (30 mm in length and 5 mm in width Figure S8c, Supporting Information) with 100 layers. The test length was set to 10 mm and the width and thickness of each sample was measured prior to the test to calculating its modulus. The test period was set to 10 min at a frequency of 1 Hz and 0.1% strain. The 3‐point bending tests were carried out with a custom‐built flexural test fixture^[^ ^37^ ^]^ at ambient conditions. The thickness of the printed sample was measured by optical microscope picture of sample fractured within liquid nitrogen. Tests were performed at a strain rate of 1.8 × 10^−3^ s^−1^ until failure. An average of six specimens were tested for each composite formulation. Thermogravimetric analysis (TGA) was performed using TGA4000 (PerkinElmer) under air environment with a heating program from 50–600 °C, at the rate of 20 °C min^−1^.

Time‐of‐flight secondary ion mass spectrometry (ToF‐SIMS) was carried out using a 3D OrbiSIMS (Hybrid SIMS)^[^ ^41^ ^]^ instrument from IONTOF GmbH (Muenster, Germany). Each secondary positively charged ion spectra was acquired in delayed extraction mode using a 30 keV Bi_3_^+^ primary ion beam delivering 0.3 pA. For the surface spectra, the primary ion beam was raster scanned over different areas with the total dose kept under the static limit of 10^13^ ions cm^−2^. The depth profiling data was acquired using a dual‐beam fashion by raster scanning the primary ion beam over regions of 300 × 300 µm^2^ at the center of a 500 × 500 µm^2^ crater formed using a 20 keV Ar_2000_^+^ ion beam delivering 5 nA. The analysis was performed in the “non‐interlaced” mode with a low‐energy (20 eV) electron flood gun employed to neutralize charge build up. One sputter frame was performed per cycle and the pause time per level was set 0.5 s. The ToF analyzer was set with 200 µs cycle time, resulting in a mass range between 0 and 3493 mass units. All 3D intensity maps were produced using the simsMVA software.^[^ ^42^ ^]^ Material intensities were normalized by total ion counts to correct for topographic features and the final 3D representations were created by combining isosurfaces ranging from 40% to 90% of the maximum intensity for each ion. Multivariate analysis was carried out using masses as the variables and each spectrum in the depth profile sequence as the observations. For dataset, Surface Lab 7.1 (IONTOF GmbH) was used to perform an automated peak search on the total spectra restricted only to peaks with intensity higher than 100 counts and masses between 30 u and 300 u. Dead‐time corrected peak areas were then exported for each pixel of all mapping datasets. NMF was performed using the simsMVA software^[^ ^42^ ^]^ using the stitch function to perform matrix augmentation and create a single matrix containing all (stitched) depth level maps and the reference images, enabling the entire dataset to be processed as a single matrix with pixels in rows and peak intensities in columns. The insertion of rows containing measurements reference materials has proven to be an effective way to identify mixed materials in an unsupervised fashion. More details of the methodology can be found in previous work.^[^ ^38^ ^]^ Initial conditions were determined by principal component analysis (PCA) and prior to NMF, data was Poisson scaled to account for heteroscedasticity.^[^ ^43^ ^]^ After 500 iterations, the analysis yielded the spatial intensity distribution of 2 endmembers with groups of secondary ion peaks that shared the same intensity spatial distribution, relating to inks A and B (Figure S4, Supporting Information). The endmember intensity maps were normalized by total intensity and smoothed using a Gaussian kernel with standard deviation of 0.5 pixel.

Glass transition point of the pure samples were measured by differential scanning calorimetry (PerkinElmer DSC 8000) with standard aluminum pan (PerkinElmer). The temperature range was −85 to 200 °C with 5 °C min^−1^ heating speed and nitrogen protection (20 mL min^−1^).

### Bacterial Biofilm Formation

*P. aeruginosa* PAO1 (Washington sub‐line) labelled with mCherry (pMMR) and *S. aureus* SH1000 labelled with GFP (pBK‐miniTn7‐egfp) were routinely grown on either LB (Luria‐Bertani, Oxoid, UK) agar plates at 37 °C or in broth at 37 °C with 200 rpm. After overnight incubation, bacteria were pelleted by centrifugation at 9500 rpm for 5 min and resuspended in RPMI 1640. Samples for biofilm attachment assessment were UV sterilized for 10 min prior to use. Bacteria were diluted in RPMI‐1640 to an OD600 = 0.01 and incubated with samples for 72 h at 37 °C and shaken at 60 rpm. Samples were washed 3 times with 10 mL of phosphate buffer saline for 5 min on a rocking platform at 60 rpm before blotting and air drying. Samples were imaged by confocal microscopy using a Carl Zeiss LSM 700 laser scanning confocal microscope fitted with 488 and 555 nm excitation lasers and a 10×/NA 0.3 objective. Images were acquired using ZEN2009 imaging software (Carl Zeiss). Bacterial biofilm surface coverage was quantified using Image J 1.44 software (National Institutes of Health, USA) and Comstat B.^[^ ^44^ ^]^

### Cell Response Experiments

Cell culture medium was prepared by adding 10% v/v of Fetal Bovine Serum (Sigma‐Aldrich, UK), 2 mm.l‐glutamine (Sigma‐Aldrich, UK) and 100 U mL^−1^ penicillin, 0.1 mg mL^−1^ streptomycin, and 0.25 µg mL^−1^ amphotericin B (Sigma‐Aldrich, UK) to Dulbecco's Modified Eagle Medium (DMEM, Sigma‐Aldrich, UK). The samples (0.7 × 0.7 × 1.5 cm) were sterilized by UV radiation for 10 min (UVP, Upland CA, USA, Cambridge, Black‐Ray XX‐15L UV bench Lamp). After sterilization, specimens were washed three times for 5 min each with phosphate buffered saline (PBS, Sigma‐Aldrich).

*Extract cytotoxicity test*:^[^ ^45^ ^]^ The samples were placed in 48 well plate and 400 µL of cell culture media was added to each sample. The extracted media were collected after 1 day, 3 days, 5 days, and 8 days. At each time point 200 µL of fresh media were collected and substituted with 200 µL of fresh media. Immortalized NIH 3T3 mouse embryonic fibroblast cells (3T3s) (passage 60) were seeded in 96‐well plate at a density of 5000 cells/well (100 µL). At an 80% confluency, the cell culture media were substituted to extract media of all time point and incubated for 24 h at 5% CO_2_, 37 °C, according to the ISO standard 10993–5:200(E). Cells cultured in cell culture media were considered as a control. Lactate dehydrogenase assay (LDH Assay kit, Thermo Scientific) and PrestoBlue assay (Invitrogen) were used to test the cytotoxicity (Figure S3, Supporting Information) of the extract media and the cell viability (Figure S4, Supporting Information), respectively. Both tests were performed according to the manufacturing protocols. Briefly, the LDH activity was measured by reading the absorbance at 490 nm (subtracted to the 680 nm) by Spectrofluorometer (Tecan Infinite M200 microplate reader). The results were compared to the maximum LDH activity, where 10 µL of Lysin Buffer (10×) was added to the cells for 30 min before performing the test and the spontaneous LDH activity, where cells were growth in normal media. PrestoBlue solution was diluted 1:10 in cell culture media and added to the microplate wells. After 45 min the fluorescence intensity of the solution, which was proportional to the cell metabolic activities, was measured at an excitation/emission wavelength of 560/590 nm, respectively, and each value was subtracted to the blank sample (media without cells).

*Cell Attachment Test*: The samples were placed in a 24‐well plate and 1 mL of cell culture media was added for 24 h. 3T3 cells were seeded over materials surfaces at a concentration of 80 000 cells/well (0.5 mL). After 24 h, samples were transferred to a new plate. The test was performed on day 1, 3, 5, and 7. For each time point, LIVE/DEAD Kit (Invitrogen, UK) was performed. Calcein AM (2.5 *μ* m) and Ethidium homodimer‐1 (5 *μ* m) were added and samples incubated for 30 min at 37 °C at 5% CO_2_. Images in green and red channels were taken by fluorescence microscope (Lumen Dynamics Leica DMIRB, USA equipped with X‐Cite Series 120 Fluorescence Illuminator, Excelitas Technologies).

### Finite Element Based Optimization

A nonlinear FE model containing 150 × 3 quadrilateral elements was used for predicting the deformation profile and angle of an MM‐IJ3DP printed cantilever containing a hinge of length of 4 mm. In each region, the material was assumed to be isotropic and linear elastic.^[^ ^46^, ^47^ ^]^ The elastic modulus measured by DMA was used and the Poisson's ratio here was estimated to be 0.4.^[^ ^48^ ^]^

During the optimization process, it was assumed that the storage modulus measured in a DMA test at 1 Hz and 0.1% strain was equal to the bending modulus in the elastic region. This model was applied to the cantilever test and the predicted deformation profile fitted the experimental data. The symmetry during the three‐point bending allowed a half model of the simply supported beam to be considered for GA optimization. A GA was employed to find the optimized spatially dependent material composition within the device for an intended objective (that would not be achievable with a homogenous material under the same loading conditions). The deformation profile target (*U*_des_) was taken from a pool of FE displacement fields generated from solutions for a simply supported beam with a randomly allocated material composition for each element and where the load applied at the centre was randomly picked to be within the range 1–3 N. In this work, the FE model of the beam was constructed from 20 × 2 quadrilateral elements (21 × 3 FE nodes, i.e., *n* = 63). Therefore, the desired deformation was described by defining displacement values of 21 × 3 control points (associated with the 21 × 3 FE nodes) on the desired deformation profile (*U*_des_). This model did not explicitly tackle shear locking and other similar pathologies. However, an analysis of a beam of rigid material with the same dimensions as this sample led to a reasonable expectation of up to 5% error, a figure which was considered acceptable for the proof of concept. A fixed population size of 200 chromosomes was set. The number of parameters in a candidate chromosome was equal to the number of quadrilateral elements, that is, 20. Each parameter had four bits allowing selection of 16 different material compositions ranging from A0 to A100 for each element, that is, the total number of bits in a chromosome was 20 × 4 = 80. Mutation rate was set to 0.002. A two‐point cross over algorithm was used. Within GA, an initial population of 200 designs was randomly generated and the fitter designs (as evaluated by the objective function specified in the main text) were selected to breed a new generation of designs (Figure S9, Supporting Information). In each iteration, the new generation of designs was created by randomly exchanging material composition characteristics within pairs of selected designs from the old generation. At this point the deformation of the designed beam under the prescribed load was converged to the defined deformation profile with the value of objective function converging to 4 × 10^−3^ mm. From Figure S9, Supporting Information, it can be seen that the objective (cost function) converged before reaching the number of maximum iterations. It can be seen that the average objective for the population was reduced from 6 × 10^−6^ to 0.5 × 10^−6^ through the GA optimization process.

### Statistical Analysis

Statistical analysis was performed using Graphpad Prism 8.0.1. All the bar plots and scatter plots were presented as mean ± standard deviation (SD) and had at least three repeats unless specifically mentioned. The tests of statistically significant difference (when *p* ≤ 0.05) were performed through a one‐way ANOVA with a post hoc Tukey test.

## Conflict of Interest

The authors declare no conflict of interest.

## Author Contributions

The manuscript was written with contributions of all authors. The majority of the experimental work was carried out by Y.H. and B.B. Support for 3D printing was provided by R.W., C.T., J.L. J.F.D. and A.H. conducted the bacterial biofilm assays overseen by P.W. and M.R.A. Cytotoxicity experiments were conducted by E.P. under the supervision of F.R. Chemical characterization and materials understanding were performed by G.F.T. and supported by C.R., R.H., D.I., and M.R.A. Custom flexural fixture was developed by G.C. and D.D.F., and data analysis was supported by G.C. and D.D.F. Modelling and optimization were performed by M.A. and overseen by I.A. The work was conceived and organized by M.R.A. and R.W.

## Supporting information

Supporting Information

## Data Availability

The data that support the findings of this study are available from the corresponding author upon reasonable request.

## References

[1] H. M. Rostam , L. E. Fisher , A. L. Hook , L. Burroughs , J. C. Luckett , G. P. Figueredo , C. Mbadugha , A. Latif , L. Kammerling , M. Day , K. Lawler , D. Barrett , S. Elsheikh , M. Ilyas , D. A. Winkler , M. R. Alexander , A. M. Ghaemmaghami , Matter (Cell Press) 2020, 2, 1564.

[2] A. L. Hook , C.‐Y. Chang , J. Yang , J. Luckett , A. Cockayne , S. Atkinson , Y. Mei , R. Bayston , D. J. Irvine , R. Langer , D. G. Anderson , P. Williams , M. C. Davies , M. R. Alexander , Nat. Biotechnol. 2012, 30, 868875. 10.1038/nbt.2316 PMC3796337 22885723

[3] Y. Mei , K. Saha , R. Bogatyrev S , J. Yang , A. L. Hook , I. Kalcioglu , S. Cho , M. Mitalipova , N. Pyzocha , F. Rojas , K. J. Van Vliet , M. C. Davies , M. R. Alexander , R. Langer , R. Jaenisch , D. G. Anderson , Nat. Mater. 2010, 9, 768. 20729850 10.1038/nmat2812 PMC3388774

[4] C. Vallieres , A. L. Hook , Y. He , V. Cuzzucoli Crucitti , G. Figueredo , C. R. Davies , L. Burroughs , D. A. Winkler , R. D. Wildman , D. J. Irvine , M. R. Alexander , S. V. Avery , Sci. Adv. 2020, 6, eaba6574. 32548270 10.1126/sciadv.aba6574 PMC7274803

[5] N. Jeffery , K. Kalenderski , J. Dubern , A. Lomiteng , M. Dragova , A. Frost , B. Macrae , A. Mundy , M. Alexander , P. William , D. Andrich , Eur. Urol. Suppl. 2019, 18, e377.

[6] B. Derby , Annu. Rev. Mater. Res. 2010, 40, 395.

[7] B.‐J. de Gans , P. C. Duineveld , Schubert , Adv. Mater. 2004, 16, 203.

[8] M. Mitchell , An Introduction to Genetic Algorithms, MIT press, 1998.

[9] D. Simon , Evolutionary Optimization Algorithms: Biologically‐Inspired and Population‐Based Approaches to Computer Intelligence, John Wiley & Sons, Hoboken, NJ 2013.

[10] A. Skylar‐Scott M , J. Mueller , W. Visser C , J. A. Lewis , Nature 2019, 575, 330. 31723289 10.1038/s41586-019-1736-8

[11] D. Han , C. Yang , N. X. Fang , H. Lee , Addit. Manuf. 2019, 27, 606.

[12] D. Raviv , W. Zhao , C. McKnelly , A. Papadopoulou , A. Kadambi , B. Shi , S. Hirsch , D. Dikovsky , M. Zyracki , C. Olguin , R. Raskar , S. Tibbits , Sci. Rep. 2014, 4, 7422. 25522053 10.1038/srep07422 PMC4270353

[13] Q. Ge , H. J. Qi , M. L. Dunn , Appl. Phys. Lett. 2013, 103, 131901.

[14] Y. He , R. Foralosso , F. Trindade G , A. Ilchev , L. Ruiz‐Cantu , E. A. Clark , S. Khaled , R. J. M. Hague , C. J. Tuck , F. R. A. J. Rose , G. Mantovani , D. Irvine , C. J. Robert , R. D. Wildman , Adv. Ther. 2020, 3, 1900187.

[15] F. Zhang , E. Saleh , J. Vaithilingam , Y. Li , C. J. Tuck , R. J. M. Hague , R. D. Wildman , Y. He , Addit. Manuf. 2019, 25, 477.

[16] A. Hosny , S. J. Keating , J. D. Dilley , B. Ripley , T. Kelil , S. Pieper , D. Kolb , C. Bader , A. Pobloth , M. Griffin , R. Nezafat , G. Duda , E. A. Chiocca , J. R. Stone , J. S. Michaelson , M. N. Dean , N. Oxman , J. C. Weaver , 3DP Addit. Manuf. 2018, 5, 103.

[17] C. Bader , D. Kolb , J. C. Weaver , S. Sharma , A. Hosny , J. Costa , N. Oxman , Sci. Adv. 2018, 4, eaas8652, 29854949 10.1126/sciadv.aas8652 PMC5976266

[18] Y. He , B. Begines , J. Luckett , J. F. Dubern , A. L. Hook , E. Prina , F. R. A. J. Rose , C. J. Tuck , R. J. M. Hague , D. J. Irvine , P. Williams , M. R. Alexander , R. D Wildman , Biorxiv 2020, 10.1101/2020.06.30.180596. PMC7613459 35033903

[19] J. W. Costerton , P. S. Stewart , E. P. Greenberg , Science 1999, 284, 1318. 10334980 10.1126/science.284.5418.1318

[20] S. S. Magill , J. R. Edwards , W. Bamberg , Z. G. Beldavs , G. Dumyati , M. A. Kainer , R. Lynfield , M. Moloney , L. MacAllister‐Hollod , J. Nadle , S. M. Ray , D. L. Thompson , L. E. Wilson , S. K. Fridkin , N. Engl. J. Med. 2014, 370, 1198. 24670166 10.1056/NEJMoa1306801 PMC4648343

[21] D. K. Lee , J. Hepatobiliary , Pancreatic Sci. 2009, 16, 628.

[22] Z. Zhou , Q. Yao , L. Li , X. Zhang , B. Wei , L. Yuan , L. Wang , Med. Sci. Monit. Int. Med. J. Exp. Clin. Res. 2018, 24, 6934. 10.12659/MSM.911770 PMC6178870 30269152

[23] J. A. Inzana , R. P. Trombetta , E. M. Schwarz , S. L. Kate , H. A. Award , Eur. Cells Mater. 2015, 30, 232 10.22203/ecm.v030a16 PMC4663047 26535494

[24] P. R. Shankar , Arch. Pharm. Pract. 2016, 7, 110.

[25] K. Adlington , N. T. Nguyen , E. Eaves , J. Yang , C. Chang , J. Li , A. L. Gower , A. Stimpson , D. G. Anderson , R. Langer , M. C. Davies , A. L. Hook , P. Williams , M. R. Alexander , D. J. Irvine , Biomacromolecules 2016, 17, 2830. 27461341 10.1021/acs.biomac.6b00615 PMC6464089

[26] P. Brouqui , M. C. Rousseau , A. Stein , M. Drancourt , D. Raoult , Antimicrob. Agents Chemother. 1995, 39, 2423. 8585720 10.1128/aac.39.11.2423 PMC162959

[27] E. A. Salvati , R. P. Robinson , S. M. Zeno , B. L. Koslin , B. D. Brause , P. D. Wilson , J. Bone Jt. Surg., Am. Vol. 1982, 64, 525. 7068695

[28] ISO 10993‐5:2009. Biological evaluation of medical devices – Part 5: Tests for in vitro cytotoxicity, International Organization for Standardization, Geneva 2009.

[29] M. A. Wagner , J. L. Huang , P. Okle , J. Paik , R. Spolenak , Mater. Des. 2020, 191, 108643.

[30] Y. Mao , K. Yu , M. S. Isakov , J. Wu , M. Dunn , H. J. Qi , Sci. Rep. 2015, 5, 13616. 26346202 10.1038/srep13616 PMC4562068

[31] Q. Ge , C. K. Dunn , H. J. Qi , M. L. Dunn , Smart Mater. Struct. 2014, 23, 09400.

[32] M. P. Bendsøe , Struct. Optim. 1989, 1, 193.

[33] M. Zhou , G. I. N. Rozvany , Comput. Methods Appl. Mech. Eng. 1991, 89, 309.

[34] Y. M. Xie , G. P. Steven , Comput. Struct. 1993, 49, 885.

[35] O. M. Querin , G. P. Steven , Y. M. Xie , Eng. Comput. 1998, 15, 1031.

[36] M. Y. Wang , X. Wang , D. Guo , Comput. Methods Appl. Mech. Eng. 2003, 192, 227.

[37] G. Y. H. Choong , A. Canciani , D. S. A. De Focatiis , Polym. Test. 2020, 85, 106430.

[38] G. F. Trindade , M. L. Abel , C. Lowe , R. Tshulu , J. F. Watts , Anal. Chem. 2018, 90, 3936. 29488747 10.1021/acs.analchem.7b04877

[39] M. K. Passarelli , J. Wang , A. S. Mohammadi , R. Trouillon , I. Gilmore , A. G. Ewing , Anal. Chem. 2014, 86, 9473. 25137365 10.1021/ac501228x PMC4188270

[40] M. P. Seah , Surf. Interface Anal. 2002, 33, 950.

[41] M. K. Passarelli , A. Pirkl , R. Moellers , D. Grinfeld , F. Kollmer , R. Havelund , C. F. Newman , P. S. Marshall , H. Arlinghaus , M. R. Alexander , A. West , S. Horning , E. Niehuis , A. Makarov , C. T. Dollery , I. S. Gilmore , Nat. Methods 2017, 14, 1175. 29131162 10.1038/nmeth.4504

[42] G. F. Trindade , M. L. Abel , J. F. Watts , Chemom. Intell. Lab. Syst. 2018, 182, 180.

[43] M. R. Keenan , V. S. Smentkowski , Surf. Interface Anal. 2016, 48, 218.

[44] A. Heydorn , A. T. Nielsen , M. Hentzer , C. Sternberg , M. Givskov , B. K. Ersboll , S. Molin , Microbiology 2000, 146, 2395. 11021916 10.1099/00221287-146-10-2395

[45] M. Pusnik , M. Imeri , G. Deppierraz , A. Bruinink , M. Zinn , Sci. Rep. 2016, 6, 20854. 26861591 10.1038/srep20854 PMC4748257

[46] M. W. Barclift , C. B. Williams , Int. Solid Freeform Fabrication Symp. 2012, 6–8.

[47] A. Kęsy , J. Kotliński , Arch. Civ. Mech. Eng. 2010, 10, 37.

[48] J. H. Lee , S. S. Lee , J. D. Chang , M. S. Thompson , D. J. Kang , S. Park , S. Park , Sci. World J. 2013, 2013, 930798.

