# Supplementary material for: Exploiting Generative Design for 3D Printing of Bacterial Biofilm Resistant Composite Devices
Source: Adv Sci (Weinh). 2021 May 29;8(15):2100249. doi: 10.1002/advs.202100249 (PMC8336490; doi:10.1002/advs.202100249)
Supplement: Supplementary file 1 — Supporting Information [file ADVS-8-2100249-s001.pdf]

© 2021 Wiley-VCH GmbH

ADVANCED SCIENCE Open Access

Supporting Information

for *Adv. Sci.,* DOI: 10.1002/advs.202100249

Exploiting Generative Design for 3D Printing of Bacterial Biofilm Resistant Composite Devices

Yinfeng He, Meisam Abdi, Gustavo F. Trindade, Belén Begines, Jean-Frédéric Dubern, Elisabetta Prina, Andrew L. Hook, Gabriel Y. H. Choong, Javier Ledesma, Christopher J. Tuck, Felicity R. A. J. Rose, Richard J. M. Hague, Clive J. Roberts, Davide S. A. De Focatiis, Ian A. Ashcroft, Paul Williams, Derek J. Irvine, Morgan R. Alexander, Ricky D. Wildman

Supplementary Materials for

Exploiting Generative Design for 3D Printing of Bacterial Biofilm Resistant Composite Devices

Yinfeng He, Meisam Abdi, Gustavo F. Trindade, Belén Begines, Jean-Frédéric Dubern, Elisabetta Prina, Andrew L. Hook, Gabriel Y. H. Choong, Javier Ledesma, Christopher J. Tuck, Felicity R. A. J. Rose, Richard J. M. Hague, Clive J. Roberts, Davide S. A. De Focatiis, Ian A. Ashcroft, Paul Williams, Derek J. Irvine, Morgan R. Alexander, Ricky D. Wildman

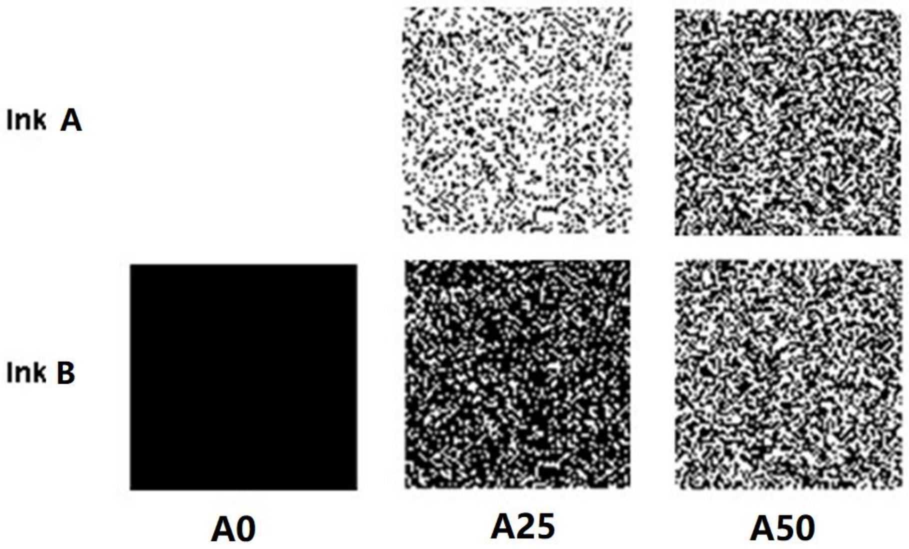

Figure S1: Examples of sub-pattern bitmaps for ink A and B are shown above, compositions of A0, A25 and A50 were chosen as exemplar and a simple square patten was used, where every black pixel represent the correlated ink droplet printed.

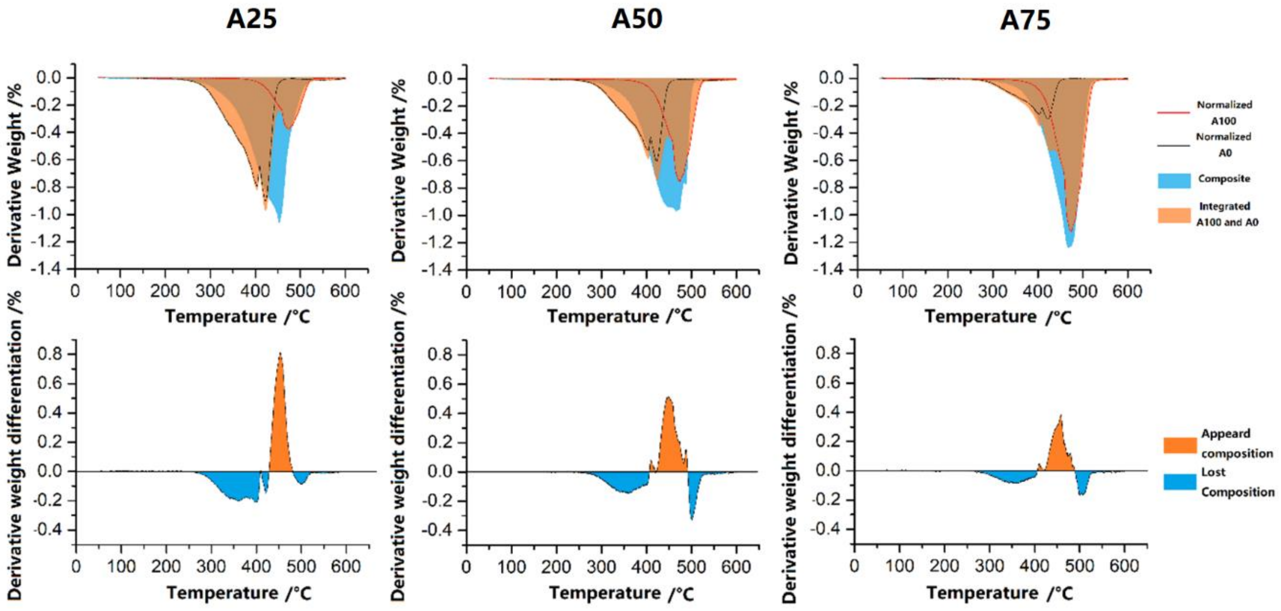

Figure S2: The comparison of derivative TGA curves for samples A25, A50 and A75. The upper row compares the addition of curves for A0 and A100 at proportions equivalent to A25, A50 and A75, with the curves obtained for A25, A50 and A75. The bottom row shows the differences where blue represents signals that are not present in the A25, A50 and A75 composites curves and orange represents signals that appear only in the composites samples.

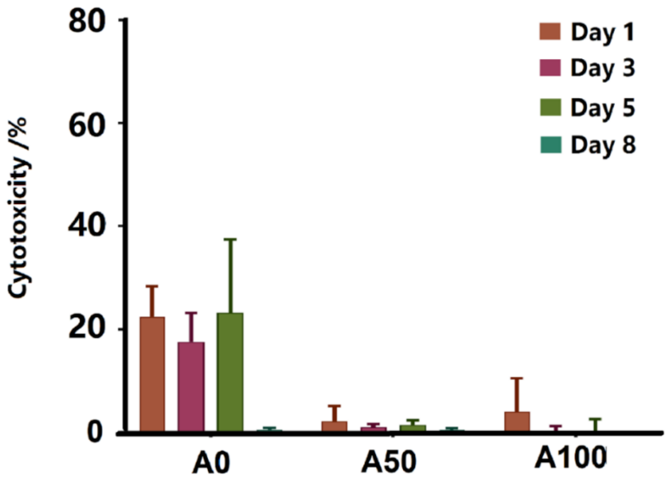

Figure S3: Extract cytotoxicity (%) comparing the cytotoxicity of a selection of printed composites (A0, A50 and A100) on different days (day 1-8). At each time point, conditioned medium extracts (media previously incubated with printed samples for 1 day, 3 days, 5 days and 8 days) were collected and transferred to wells containing a monolayer of immortalized NIH 3T3 mouse embryonic fibroblasts for 24 hours; the LDH assay was then carried out using the supernatant to determine cell cytotoxicity. The data are presented as mean ± standard deviation, n = 3.

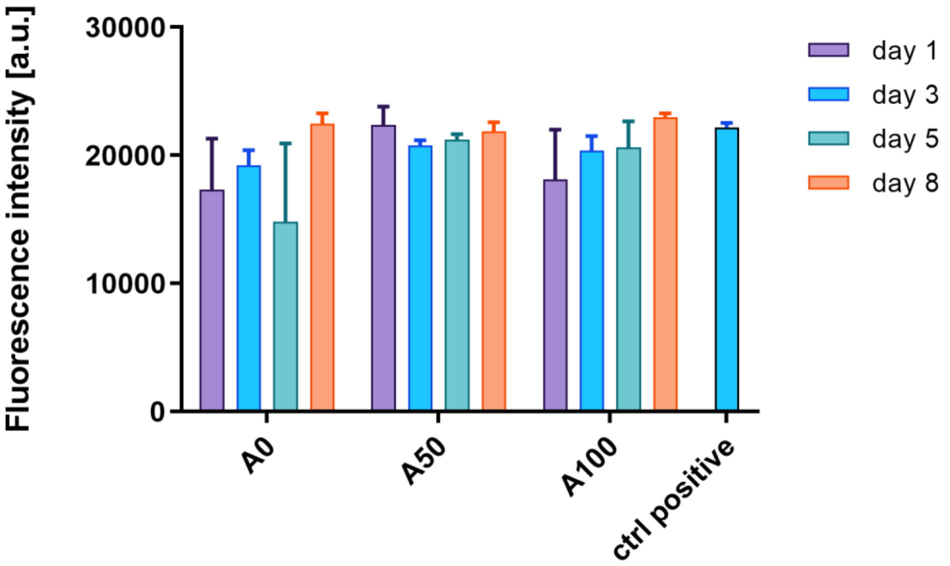

Figure S4: Cumulative extract cell viability measured by PrestoBlue. At each time point, conditioned medium extracts were collected and transferred to wells containing a monolayer of immortalized NIH 3T3 mouse embryonic

fibroblasts for 24 hours . The error bars equal ± one standard deviation unit, n = 3. Statistically significant differences (*p ≤0.05) were sought using a one-way ANOVA with post-hoc Tukey's test, but none were found with respect to the control.

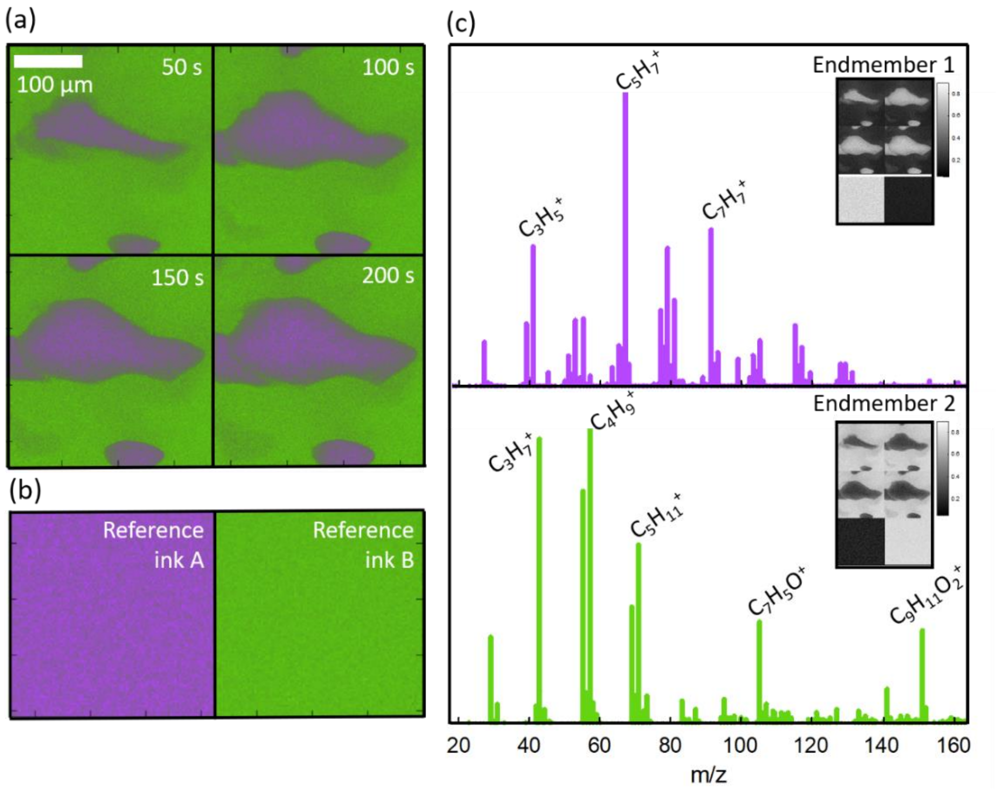

Figure S5: Output of non-negative matrix factorisation analysis. (a) Overlay of endmembers intensity distribution of ToF-SIMS mapping data at different sputter times. (b) Endmember intensities for reference samples printed by pure inks A and B. (c) Peaks comprising endmembers 1 (ink A, purple) and 2 (Ink B, green).

Table S1: Thermal properties of representative formulations measured by TGA, ^{0}Td is the temperature where the onset of decomposition was observed, ${}^{max}T_d$ is the point where a peak is observed in the derivative curve of the TGA test, and each temperature represents one observed peak on the curve.

**Formulation**	**Temperature**	
	^{o}Td [ºC]	^{max}Td [ºC]
A0	317	397/414
A25	348	436/453
A50	368	449/462/480
A75	391	466
A100	422	468

Development of ink formulations and printing strategy for MM-IJ3DP

Ink A formed a rigid polymer after printing and consisted of 50 v/v% ethylene glycol dicyclopentenyl ether acrylate (EGDPEA) and 50 v/v% tricyclo decanedinmethanol diacrylate (TCDMDA) as structural monomers. Both EGDPEA and TCDMDA have been confirmed to inhibit *P. aeruginosa* and *S. aureus* biofilm formation when polymerized [1,2]. Ink B contained 60 v/v% 2-ethylhexyl acrylate (EHA) and 40 v/v% hydroxybutyl acrylate (HBA) and was formulated to produce a flexible material. When selecting the components for ink B, the glass transition point (T_g) of the polymers was also taken into consideration with a guide that those with a T_g lower than room temperature tend to be flexible at that temperature[22], and those higher are more rigid (Supplementary Table S2). Poly-EHA is a candidate (T_g ~ -70°C) for inhibition of biofilm formation of both bacterial strains[2], but not suitable for IJ3DP due to its viscosity, reactivity and volatility. Consequently, we blended this material with HBA to aid the printing, since HBA has a similar T_g and preliminary inkjet printing trials had shown it to be printable. 2,2 Dimethoxy-2-phenylacetophenone was chosen as the photoinitiator as it was soluble in both ink formulations. The rheology of the inks was determined, and the printing parameters tailored to ensure reliable printing (Supplementary Figure S6/S7, Table S3). Each layer was divided into two sub-patterns (A and B) and assigned to different printheads for ink A and B (Supplementary Figure S1). A pseudorandom pixel deposition strategy using Mathematica (v10.4) was used to create a different pattern for each layer, whilst keeping the fraction of A and B constant. The pseudorandom co-printing strategy was tested using two coloured commercial inks and created a simple gradation in colour. Thus, printing a whole layer consisted of four steps: 1) printing of ink A; 2) application of UV light for pinning (1 s); 3) printing of ink B; 4) application of UV light for curing (25 s). From this point onwards, all the polymer samples were named as ‘A’ plus number (1-100) representing the ratio of ink A in the composite, e.g. A25 (25% pixels filled with ink A and 75% filled with ink B).

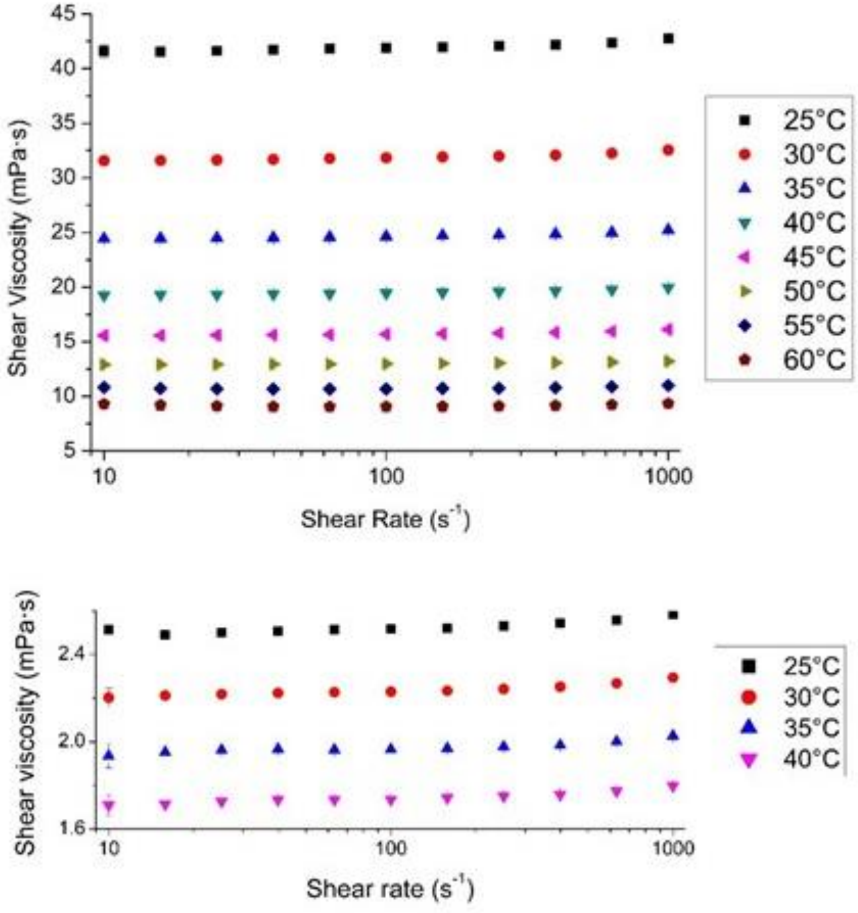

Figure S6: Viscosity of ink A (top) and ink B (bottom) are measured using a cone plate system, in a shear rate range from 10 s^{-1} to 1000 s^{-1}, the temperatures were chosen from room temperature up to the printing temperature with an interval of 5°C , the data are shown as mean±standard deviation, n = 3.

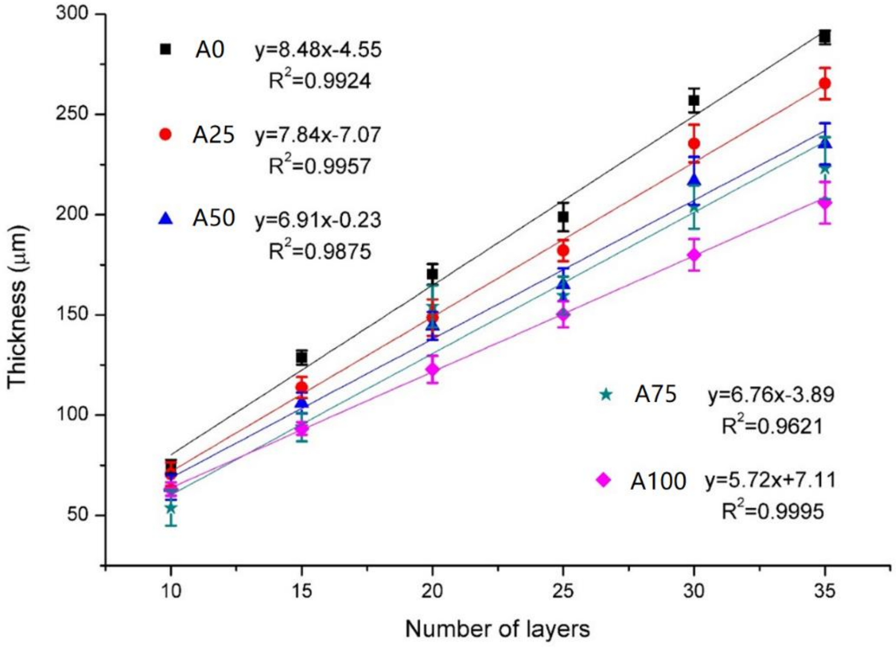

Figure S7: The average layer thickness of the printed composites were estimated by applying simple linear regression to measured specimens height consisted of 10 to 35 layers. The data is presented as mean ± standard deviation, n = 3

Table S2: Glass transition point of the homopolymer prepared from each chosen monomer. The sample were prepared by UV polymerization in a vial and tested by DSC at a heating rate of 5°C/minutes and temperature range of -85°C to 200°C

**Formulation**	**Glass transition point [°C]**
Poly-TCDMDA	160°C
Poly-EGDPEA	19°C
Poly-EHA	-70°C
Poly-HBA	-65°C

Table S3: Printing parameters and jetting waveform of the two ink formulations (A and B) for printing on the Pixdro LP50 platform.

Inks	Pulse shape			Voltage [V]	Ink Pressure [mbar]	Droplet Spacing [µm]
	Rising Edge [µs]	Peak [µs]	Falling Edge [µs]			
A	2	4	2	90	-20	70
B	1	4	10	83	-18	70

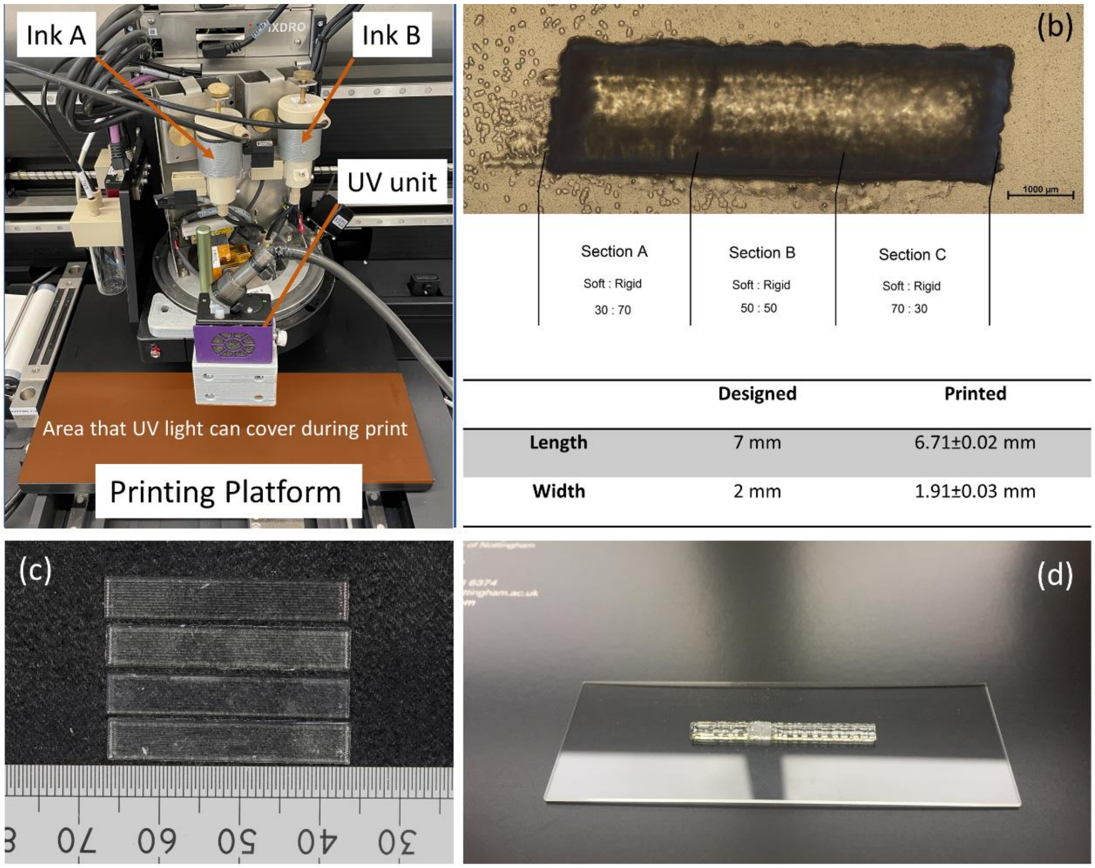

	Designed	Printed
**Length**	7 mm	6.71±0.02 mm
**Width**	2 mm	1.91±0.03 mm

Figure S8: a) Printing platform and UV illuminating area of the Pixdro LP50 printer with laboratory developed UV attachment, where the orange region shows the maximum printing area (105 mm x 297 mm). All the printing was performed at a 70µm droplet spacing (equivalent to 423.33 DPI); b) three section bar printed for in vitro bacterial attachment test, where section A contained 70 v/v% ink A, section B contained 50 v/v% ink A and section C contained 30 v/v% ink A; c) a batch of printed samples for DMA testing; d) simple beam incorporating a soft hinge, printed onto a standard glass slide (75 mm*25 mm).

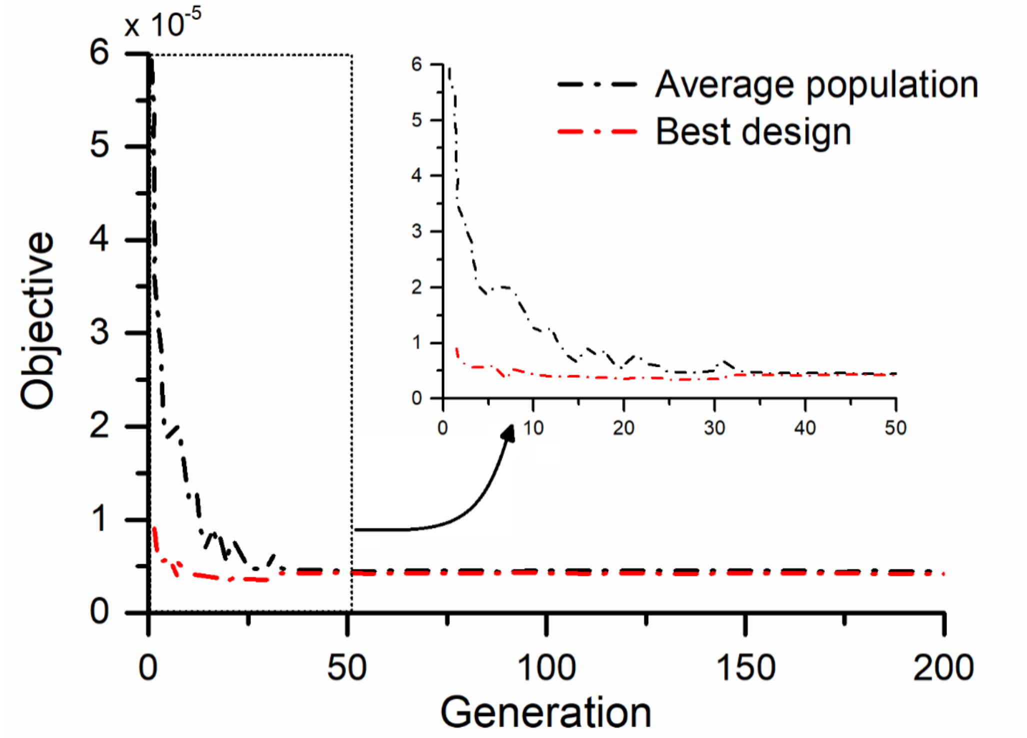

Figure S9: Generation history of objective function (average design population and best design) for the simply supported multi-material beam.
